# Stimuli-Responsive Nucleotide–Amino Acid Hybrid Supramolecular Hydrogels

**DOI:** 10.3390/gels7030146

**Published:** 2021-09-17

**Authors:** Matthew Mulvee, Natasa Vasiljevic, Stephen Mann, Avinash J. Patil

**Affiliations:** 1 Centre for Organized Matter Chemistry, School of Chemistry, University of Bristol, Bristol BS8 1TS, UK; mattmulvee@gmail.com; 2 Bristol Centre for Functional Nanomaterials, University of Bristol, Tyndall Avenue, Bristol BS8 1TL, UK; n.vasiljevic@bristol.ac.uk; 3 School of Physics, University of Bristol, Bristol BS8 1TS, UK

**Keywords:** self-assembly, nucleotide, amino acid, supramolecular hydrogels, hybrid hydrogels, stimuli-responsive

## Abstract

The ability to assemble chemically different gelator molecules into complex supramolecular hydrogels provides excellent opportunities to construct functional soft materials. Herein, we demonstrate the formation of hybrid nucleotide–amino acid supramolecular hydrogels. These are generated by the silver ion (Ag^+^)-triggered formation of silver–guanosine monophosphate (GMP) dimers, which undergo self-assembly through non-covalent interactions to produce nanofilaments. This process results in a concomitant pH reduction due to the abstraction of a proton from the guanine residue, which triggers the in situ gelation of a pH-sensitive amino acid, N-fluorenylmethyloxycarbonyl tyrosine (FY), to form nucleotide–amino acid hybrid hydrogels. Alterations in the supramolecular structures due to changes in the assembly process are observed, with the molar ratio of Ag:GMP:FY affecting the assembly kinetics, and the resulting supramolecular organisation and mechanical properties of the hydrogels. Higher Ag:GMP stoichiometries result in almost instantaneous gelation with non-orthogonal assembly of the gelators, while at lower molar ratios, orthogonal assembly is observed. Significantly, by increasing the pH as an external stimulus, nanofilaments comprising FY can be selectively disassembled from the hybrid hydrogels. Our results demonstrate a simple approach for the construction of multicomponent stimuli-responsive supramolecular hydrogels with adaptable network and mechanical properties.

## 1. Introduction

Typically, supramolecular gels are formed from the assembly of a single compound, known as a gelator, into filaments that laterally associate and entangle to produce a network that gels a solvent. Multicomponent gels formed from two or more components are an appealing extension to create more complex systems, with different functionalities and responses to stimuli, such as modulating mechanical strength or having one gelator susceptible to a particular stimulus. Furthermore, such systems are more akin to biological systems whereby the assembly of different components (e.g., nucleotides, lipids, and amino acids) must self-sort or combine to assemble into a myriad of structures. For example, the cytoskeleton of cells is made up of microtubules and actin filaments that undergo orthogonal assembly and disassembly, resulting in variable and adaptable mechanical properties. Such systems also serve as an inspiration to engineer more sophisticated self-assembled structures over different length scales [1,2,3]. The assembly of multiple gelators is complicated due to the possibility of intermixing of the components during the hierarchical organisation. The gelators can orthogonally ‘self-sort’ into separate ‘narcissistic’ filaments or co-assemble into mixed filaments [4,5,6,7,8,9]. There are several ways for the gelators to organise in hybrid filaments including alternating domains of single-components or mixing in ordered or random arrangements. Additionally, the filaments of the different gelators may self-sort or mix. Finally, the co-assembly of the gelators in certain cases can be disruptive such that the physicochemical and mechanical properties are hampered by the disordered supramolecular assembly. This often occurs through a mismatch in the alignment of the gelators such that the ordering of the intermolecular interactions is perturbed relative to how the individual gelators assemble [4].

Multicomponent hydrogels are relevant to the fabrication of more complex hydrogel scaffolds with higher informational content, for instance, the presence of multiple synergistic functional groups that stimulate tissue growth [10] or mimic the physical and chemical properties of the extracellular matrix by combining two derivatised peptides functionalised with different biologically active epitopes [11]. In the latter case, only the co-assembled gel stimulated the growth of the precursor cells into the desired structures, while the individual gelators were essentially inactive. Hybrid hydrogels are also used in optoelectronics, where self-sorted filaments can be utilised to generate bulk p-n heterojunctions [12,13,14,15,16,17]. The co-assembly of different gelators can also be utilised to tailor the mechano-chemical properties of soft materials. For example, light-sensitive multicomponent hydrogels have been prepared using stilbene and naphthalene-amino acid derivatives [18]. Irradiation of the gels with 365 nm light induced the disassembly of the stilbene-based gelator nanofibers via a trans-cis isomerisation, whereas the nanofibers formed by the amino acid gelator component were unaffected, leaving an intact hydrogel network. As opposed to “negative etching” (removing one component), “positive writing” by forming a multicomponent gel using two different proton sources to trigger the assembly of two 1,3:2,4-dibenzyldene-D-sorbitol (DBS) derivatives with different pKa values has been demonstrated [19]. The first gelator (DBS-CO_2_H) is gelled by the hydrolysis of glucono-δ-lactone (GdL) and an associated decrease in pH due to its higher pKa value. Then, the second gelator (DBS-Gly; lower pKa) is selectively gelled by the photoactivation of diphenyl iodonium nitrate (DPIN) using a mask. Doing this in a preformed gel network lowers convection and diffusion effects, thus enhancing spatial resolution. Gels with tuneable network properties are also attracting interest owing to numerous applications such as determining cargo release and affecting the mechanical properties of tissue culture scaffolds [20,21]. An elegant example is the use of the acidic interface of bola-amphiphilic hydrogelator nanofibers to catalyse the formation of a second gelator molecule, trishydrazone, that subsequently co-assembles in situ to form mixed component hydrogels [22]. 

**Figure 1:**
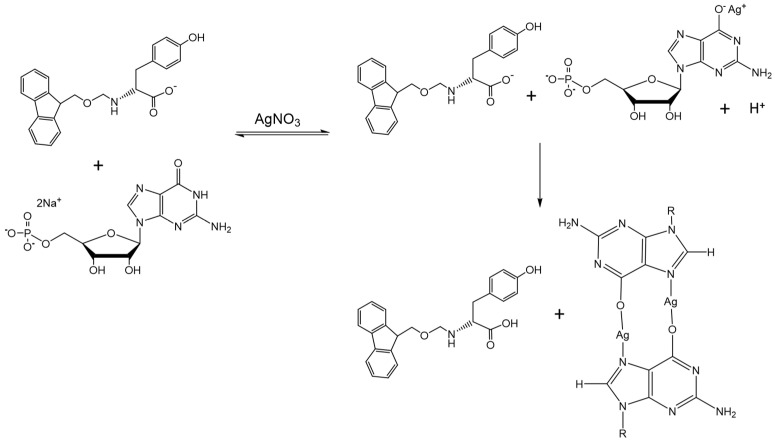
Schematic illustrating the formation of multicomponent nucleotide–amino acid hydrogels through the additions of silver ions to the mixture of FMOC-Tyrosine (FY) and guanosine monophosphate (GMP) solutions.

In this study, we utilise the coupling of a chemical signal between two gelator systems to control the orthogonal and non-orthogonal assembly modes of hybrid nucleotide–amino acid hydrogels. We show that the gelation of a nucleotide, guanosine monophosphate (GMP), can be exploited as a trigger for the in situ gelation of an amino acid derivative, N-fluorenylmethyloxycarbonyl tyrosine (FY). This is achieved by adding aqueous silver nitrate to an aqueous mixture of GMP and FY, which lowers the pH through the abstraction of a proton from the guanine residue of GMP [23,24,25,26]. The concomitant drop in pH promotes the self-assembly and gelation of FY by protonation of the amino acid carboxylate group (Figure 1). Significantly, the ratio of Ag^+^ to GMP determines the kinetics of assembly of FY such that supramolecular assembly and packing of the gelator molecules and the mechanical properties of the resulting hybrid hydrogels can be manipulated.

## 2. Results and Discussion

Single-component Ag-GMP and FY supramolecular hydrogels were produced using previously described methods and were consistent with the literature [25,27]. Nucleotide gels were formed by adding aqueous solutions of silver nitrate (50 mM or 100 mM) at an equal volume to aqueous GMP solution (50 mM) to give a final concentration of 25 mM and Ag:GMP ratios of 1:1 or 2:1, which were also used for the multicomponent hydrogels. An opaque self-supporting hydrogel was formed almost instantaneously at a ratio of 2:1, while a viscous solution that was not self-supporting after vial inversion was produced at a ratio of 1:1 (Supplementary Figure S1). Slightly turbid self-supporting FY hydrogels were prepared by dissolving FY at high pH followed by the controlled lowering of the pH below 7 using GdL to protonate the amino acid carboxylate [27,28,29]. (Figure S1a).

**Figure 2:**
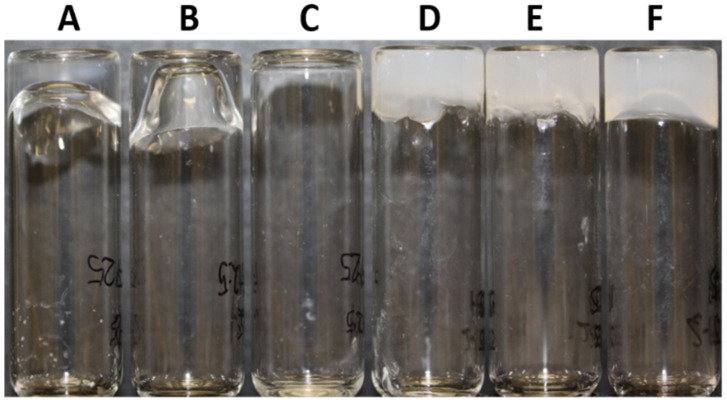
Photo of multicomponent nucleotide–amino acid hydrogels prepared by maintaining the Ag:GMP molar ratio and varying GMP:FY molar ratios. The final composition of each hydrogel is presented in Table 1.

Multicomponent gels were prepared by adding aqueous silver nitrate at equal volumes to an aqueous mixture of FY and GMP at a range of Ag:GMP:FY molar ratios (Figure 2. Refer to Table 1 for sample codes and corresponding molar ratios). The gelation behaviour was dependent on the molar ratios. For example, samples **A** and **B** gelled overnight, whereas sample **C** produced a viscous suspension. The latter was attributed to the lower Ag^+^ and GMP concentrations that were insufficient to decrease the pH below 7 and attain the critical gelation concentration, respectively. In contrast, at Ag:GMP ratios of 2:1, gelation times of several minutes (**D**) or several hours (**F**) were observed for FY concentrations of 25 mM, while sample **E** gelled instantly despite a lower FY concentration (12.5 mM) (Table 1). The lack of correlation between total gelator concentration and gelation time is consistent with the non-orthogonal assembly of the gelator molecules. Significantly, in control experiments, neither the mixing of GMP and FY nor the addition of silver nitrate to an FY solution produced hydrogels (Figure S1b), indicating that the presence of both GMP and Ag^+^ ions were required for FY self-assembly and the production of nucleotide–amino acid hybrid hydrogels.

Transmission electron microscopy (TEM) analysis of the single-component hydrogels showed the presence of a dense network of nanofilaments. FY filaments were imaged by uranyl acetate negative staining and had a flat tape morphology with diameters of 9–15 nm (Figure S2). In contrast, the Ag-GMP nanofilaments could be observed unstained and were 3–10 nm in diameter for samples prepared at 1:1 and 2:1 Ag:GMP molar ratios. Higher magnification images and EDX analysis showed that the nanofilaments were decorated with silver nanoparticles due to electron beam-induced reduction of excess silver ions (see ESI) [25]. TEM analysis of the multicomponent hybrid hydrogels also showed the presence of a highly entangled matrix of nanofilaments with dimensions comparable to that of the single-component samples (Figure S3). Notably, the viscous sample **C** showed the formation of a low-density nanofilament network, indicating that the self-association of the gelator molecules also occurred under these conditions. Although the above samples confirmed the presence of self-assembled nanostructures, a clear differentiation between the Ag-GMP and FY nanofilaments was challenging using TEM, particularly as EDX analysis could not distinguish between the different types of filaments. Therefore, we undertook extensive circular dichroism (CD) spectroscopy studies to probe the assembly of the two gelators in the multicomponent systems. 

**Figure 3:**
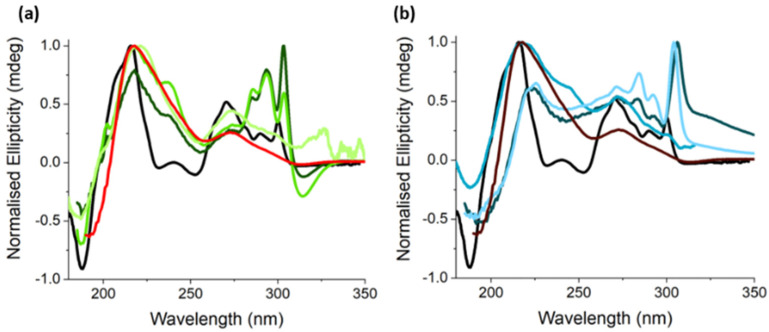
CD spectra of single-component control hydrogel samples—FY gel prepared by hydrolysis of GdL (black) or Ag-GMP gel (1:1 (red) and 2:1 (brown)). (**a**) Hydrogel samples prepared using using a 1:1 Ag:GMP molar ratio and varying FY concentrations, samples A (**―**), B (**―**), and C (**―**) and (**b**) at 2:1 Ag:GMP stoichiometry and FY varying concentrations; samples D (**―**), E (**―**), and F (**―**). Refer to Table 1.

CD spectra of aqueous FY before gelation showed peaks at ca. 210 nm, 225 nm, and 265–300 nm (Figure S4), which were consistent with previous studies which indicated that FY and structurally similar gelators assemble into worm-like micelles with supramolecular chirality at the concentrations used in our studies [4,30,31,32,33]. Upon gelation of the FY solution, the above peak positions were retained, but the ellipticity of the characteristic peaks was significantly increased (Figure 3 and Figure S4b), indicating enhanced supramolecular chirality, arising from extended filament growth and bundling [31,34]. The absorptions observed at ca. 210 nm were characteristic of n→π* transitions associated with the fluorenyl moiety, whereas absorbances between 265 and 300 were due to tyrosine and fluorenyl π→π* transitions [31,34]. Additionally, the peak at 304 nm was assigned to a π→π* transition induced by the interactions of fluorenyl groups in the supramolecular assembly [4,31,34,35]

Similarly, GMP aqueous solutions (25 mM, pH 8) exhibited relatively low ellipticity with a negative band at 196 nm arising from the chiral ribose moiety, a positive band centred at 219 nm due to an n→π* transition, and two weak negative bands at 250 and 253 nm attributed to π→π* transitions originating from the guanine chromophore (Figure S3a). These transitions were attributed to GMP monomers, dimers, and tetramer stacks, all of which give rise to a CD signal [36,37,38,39]. The Ag-GMP hydrogel spectra had a marked increase in ellipticity and showed a red-shift for the π→π* transition (Figure 3), which is indicative of overlap between the aromatic moieties contributing to exciton coupling [24,40,41,42,43].

Significantly, for the multicomponent hydrogels, the CD spectra revealed characteristic signatures associated with the control nucleotide and amino acid hydrogels, suggesting that both gelators assembled to form filaments. For the 1:1 (Ag:GMP) ratio, **A** (1:1:1), and **B** (1:1:0.5), the hydrogels exhibited a split Cotton effect with a negative and positive peak at 304 and 315 nm, respectively, which is characteristic of the extended helical arrangements of the fluorenyl groups [44,45]. The peaks were red-shifted relative to the single-component FY gel, indicating a different environment in the multicomponent system, potentially bundling with the Ag-GMP filaments. For example, tyrosine has previously been reported to intercalate between GMP stacks [46,47]. Thus, it is possible that the hybrid hydrogels consist of heterogeneous filaments. However, we note that shifts in the CD peaks of turbid samples have also been attributed to chiral scattering and therefore may not necessarily be indicative of a different assembly process when compared with the single-component samples [48]. In addition, the CD spectra of hydrogels **A** and **B** showed peaks at 275 nm, and at 285 and 293 nm associated with Ag-GMP and FY gelation, respectively. The characteristic signal at 270 nm for FY gelation was not present, which was probably due to the low concentration of FY associated with these samples. For these gels, a broad peak at short wavelengths (221 nm) was observed, which was characteristic of Ag-GMP gelation, as well as a shoulder peak at ca. 200 nm associated with FY gelation. The spilt Cotton effect was notably absent from hydrogel **C**, which was instead dominated by a high noise background due to low levels of FY filament formation under these preparation conditions. Only peaks associated with GMP were observed at shorter wavelengths for sample **C**.

For hybrid gels prepared with an Ag:GMP ratio of 2:1 and by varying FY concentrations (hydrogels **D**, **E**, **F**; Table 1), the split Cotton effect was absent for all samples. This may be related to the turbidity of the samples but may also indicate disruption of the FY assembly process. For samples **D** and **F**, there was a significant bathochromic shift of the 300 nm peak to 306 and 304 nm, respectively. Other peaks between 250 and 300 nm, associated with the fluorenyl and phenyl π→π* transitions were also observed. The peak at 270 nm was particularly prominent, which was potentially due to the lower pH achieved in these preparations, thus promoting more FY gelation [49]. Notably, in the case of hydrogel **E**, additional features originating from both the gelator molecules were observed. For instance, a negative peak at 188 nm and a shoulder at 217 nm for FY and a broad peak at 209 nm corresponding to the Ag-GMP supramolecular assembly were observed. When the concentrations of FY were the same (**D**) or higher (**F**) than GMP, the prominent peak at ca. 220 nm (n→π*) shifted to ca. 200 nm, whereas the negative feature at ca. 190 nm was absent for **D** and **F**. This Cotton effect is characteristic of helical assembly [50,51,52], and its absence further indicates disruption to the supramolecular assembly of FY. As the chiral environments probed by the CD measurements are sensitive to the electronic environments [53,54,55] and are comparable between samples, the above observations indicate significant disruption to the supramolecular organisation of the gelators in the multicomponent systems.

**Figure 4:**
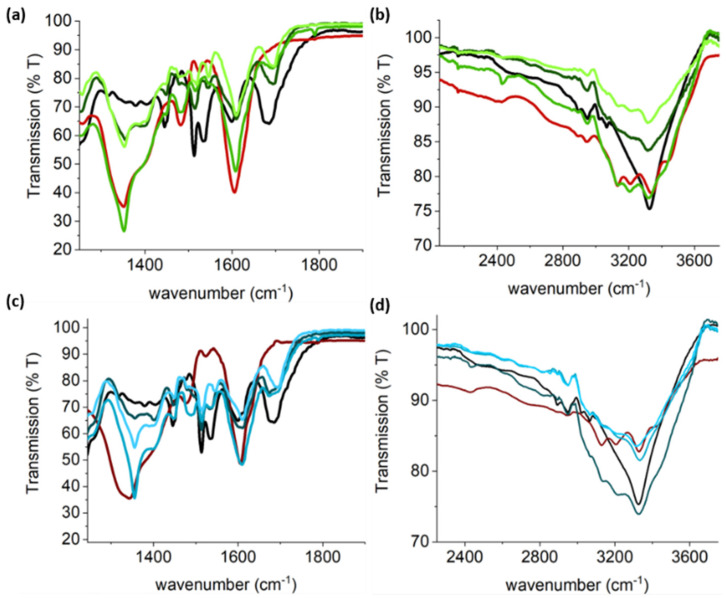
FTIR spectra of lyophilised multicomponent hydrogels. (**a**,**b**) 1:1 Ag:GMP molar ratio and varying FY concentrations, samples A (**―**), B (**―**), and C (**―**), whereas (**c**,**d**) 2:1 Ag:GMP stoichiometry and FY varying concentrations; samples D (**―**), E (**―**), and F (**―**).

Fourier transform infrared (FTIR) spectra of lyophilised samples of the single-component samples were consistent with previous reports (see ESI for detailed analysis of single-component gels, Figure S5) [4,56,57,58,59,60,61]. For the multicomponent gels prepared with a 1:1 Ag:GMP ratio (**A**, **B**, and **C**), a carbamate peak at 1693 cm^−1^ was observed for all samples (Figure 4a,b), indicating supramolecular assembly of the FY gelator. Interestingly, the enol-Ag^+^ peak featured at slightly higher wavenumbers (1608–1610 cm^−1)^ in the hybrid gels relative to the single-component Ag-GMP gels (1605–1606 cm^−1^), indicating a slight weakening of this bond, perhaps indicating some interactions between FY and GMP. This was further supported by the observation that this shift was less perturbed when the FY concentration was lower than GMP, for example in hydrogel **B**. In addition, the amide II region (ca. 1515–1550 cm^−1^) was disrupted, suggesting that the hydrogen bonding involved in GMP supramolecular assembly was affected. At higher wavelengths, the stretching bands associated with hydrogen-bonded amines of guanine were less pronounced when FY was at the same or higher concentration than GMP (Figure 4a,b). However, the 1675 cm^−1^ band characteristic of disordered carbamate arrangements was absent in the hybrid hydrogels produced at a 1:1 Ag:GMP ratio, suggesting that the slow assembly of these gels possibly facilitated more ordered hydrogen bonding. 

Similar observations were made for multicomponent hydrogels formed from a 2:1 Ag:GMP ratio and various FY concentrations (Figure 4c,d; (samples **D**, **E**, and **F**, Table 1). A broad peak was observed at 1694 cm^−1^ for all compositions and attributed to a hydrogen-bonded carbamate group. This was red-shifted relative to the 1:1 ratio gels, which was perhaps due to the lower pH achieved prior to gelation, producing a more extended hydrogen-bonded network. The peak at 1672 cm^−1^ for hydrogels **D** and **E** was attributed to disordered carbamate assembly, which was likely a consequence of the fast kinetics of gelation, as this was not observed for sample **F** that gelled on a much slower timescale. The kinetics of gelation has been shown to determine hydrogel ordering at the microscale, with slower gelation often producing a more homogenous gel network [27,62,63,64]. A larger bathochromic shift of 3–5 cm^−1^ relative to the single-component gels was observed for the enolate-Ag^+^ moiety, indicating more disruption to the GMP assembly at 2:1 Ag:GMP ratios. This was shifted to a lesser extent when the FY concentration was less than the GMP concentration, again indicating increased levels of orthogonal assembly as the FY concentration was reduced. Peaks in the amide II region (ca. 1515–1550 cm^−1^), which are associated with hydrogen-bonded amines, were more prominent for samples **D**, **E**, and **F** than for samples prepared at a 1:1 Ag: GMP ratio, which is consistent with the lower pH (increased levels of carboxylate protonation) attained in the presence of increased levels of Ag^+^ at 2:1.

The observed non-orthogonal assembly can be understood if Ag^+^ ions are associated with both FY and GMP moieties. This would perturb GMP assembly and thus also affect the pH reduction required to trigger FY gelation. Indeed, there are reports of Ag^+^ ion binding to amino acids and subsequent reduction to Ag nanoparticles [52,65,66,67,68,69]. In this regard, the hydroxyl group of tyrosine is deprotonated above ca. 10.1, and subsequent electron transfer to Ag^+^ produces Ag nanoparticles and a quinone moiety [67]. However, as the pH remains below the tyrosine pKa after the addition of GMP to FY (pH = ca. 8.5), electron transfer is not expected to occur in the hydrogel systems (see ESI for more details).

**Figure 5:**
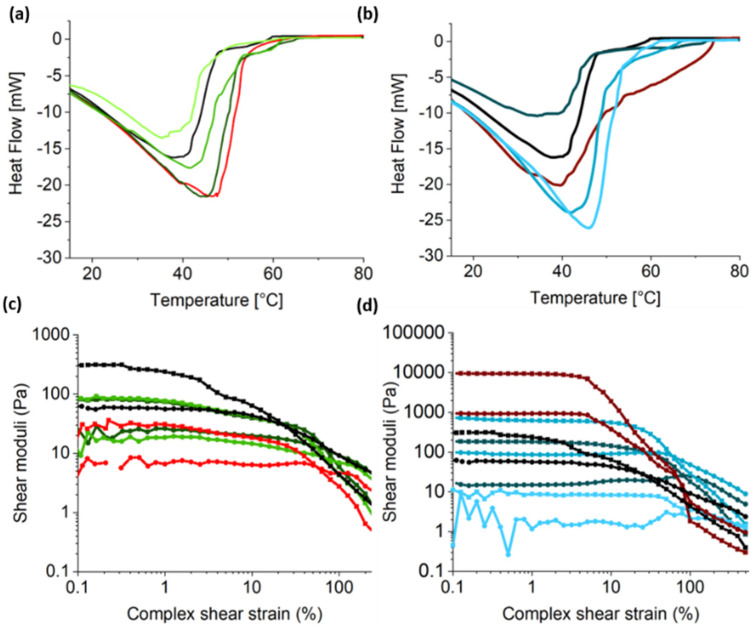
DSC thermograms of the multicomponent supramolecular hydrogels alongside single-component hydrogels at the (**a**) 1:1 and (**b**) 2:1 ratio. Rheology studies showing oscillatory amplitude sweeps at a constant frequency (1 Hz) (**c**,**d**). All gels exhibited a linear viscoelastic regime. In oscillatory experiments, ▪ refer to G’ and • refer to G”, the storage modulus and viscous modulus, respectively. For all datasets, multicomponent samples 1:1 Ag:GMP stoichiometry and varying FY concentrations; samples: A (**―**), B (**―**), and C (**―**) and at 2:1 Ag-GMP stoichiometry and vary molar ratios; samples: D (**―**), E (**―**), and F (**―**). Red lines refer to the control Ag-GMP gel samples.

The consequences of the aforementioned non-orthogonal assembly on the macroscopic properties of the hydrogels were studied using differential scanning calorimetry (DSC) and oscillatory rheology (Figure 5). The DSC thermograms displayed a broad endothermic peak for all samples, corresponding to the disruption of the non-covalent crosslinked matrix of the supramolecular hydrogel system, which facilitates the gel-to-sol transition [25,70,71,72]. The single-component FY gel showed a broad minimum at 40.1 °C with a broad shoulder in the region of 48–60 °C. In the case of the single-component Ag-GMP gels, at 1:1 (Ag:GMP), the sample showed a melting peak centred at 46.8 °C, whereas at a 2:1 ratio, the samples showed a very broad peak with a minimum at 39.7 °C and several other smaller peaks in the region of 52–74.4 °C. This indicated heterogeneity in the gelation, which may be a consequence of the relatively slow dissolution of the silver salt relative to gelation and is analogous to using mineral salts to lower the pH as a trigger for gelation. 

For the multicomponent hydrogels, the DSC observations were consistent with the non-orthogonal assembly of the two components. At a 1:1 Ag:GMP ratio and different FY concentrations, samples **A**, **B**, and **C** have a decreasing T_gel-sol_ (Table S1). This is in line with a lower total gelator concentration for **B** and **C** and therefore a lower network density. Interestingly, this relationship does not hold for the multicomponent hydrogels prepared at a 2:1 Ag:GMP ratio. For instance, two endothermic peaks were observed for hydrogels **D** and **E**, which is indicative of heterogenous gelation. This was not observed for **F**, which was presumably due to the increased homogeneity arising from the slower gelation time. In addition, the endothermic peaks for **E** are higher than the peaks observed for **D**, despite the lower total gelator concentration. This further supported non-orthogonal assembly, as the T_gel-sol_ is expected to increase with increasing gelator concentration [73]. Oscillatory amplitude sweeps at a constant frequency (1 Hz) demonstrated a linear viscoelastic region (LVR) for all the hydrogels, in which the storage (elastic) G’ moduli were roughly an order of magnitude higher than the loss (viscous) G” moduli. This indicated that an elastic solid-like nanofilament network permeated throughout a viscous solvent [25,27,34,70,74]. All samples showed crossover points with a sharp decrease in storage moduli (G’) compared to viscous moduli (G”), which were assigned to the transition from an elastic gel to a viscous fluid [25,71,75,76]. At low strain, the single-component FY gel had an elastic modulus of ca. 310 Pa and a yield strain of ca. 25%. For the 1:1 single-component Ag-GMP gel, the elastic moduli (ca. 30 Pa) was not significantly higher than the viscous moduli (ca. 7 Pa) and hence was not classified as a gel, rather a viscous solution, although a yield strain of ca. 50% was observed, as the filaments formed in the solution could still be sheared. For the single-component 2:1 Ag-GMP gel, within the LVR, the elastic modulus was ca. 9500 Pa, and the yield stress ca. 63%. In contrast, the multicomponent hydrogels prepared at a 1:1 Ag:GMP ratio and different FY concentrations were stiffer than the single-component Ag-GMP gels but noticeably less stiff than the single-component FY hydrogel. Notably, all hybrid gel samples (**A**–**E**) showed enhanced yield stresses compared to the single-component hydrogels, which could be attributed to the highly entangled network of nanofilaments originating from the self-assembly of both the nucleotide and amino acid gelators. Significantly, at both Ag:GMP ratios, the measured elastic moduli were higher when the FY concentration was lower than the GMP concentration. This could be due to the non-orthogonal assembly of the gelator molecules as inferred from the above analysis. 

**Figure 6:**
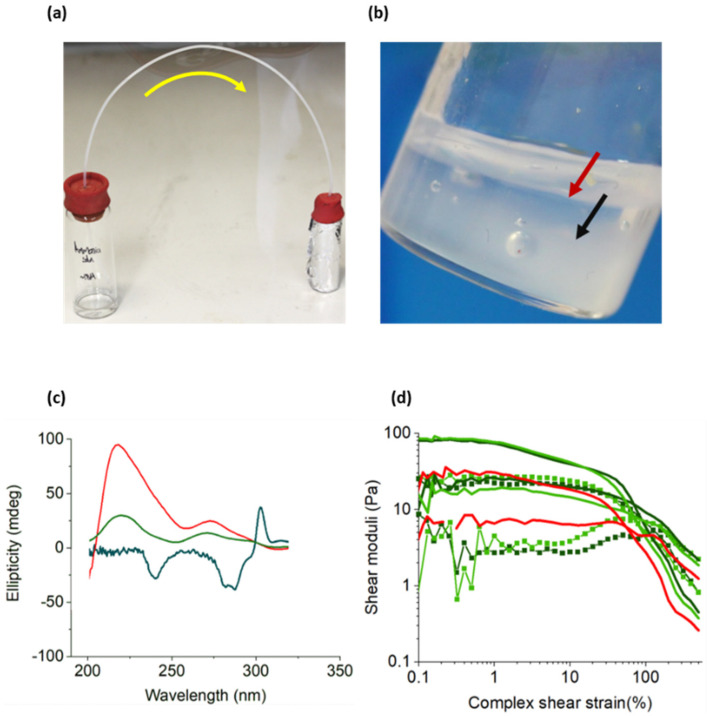
Selective disassembly of multicomponent hydrogels. (**a**) Photo of set up to raise pH whereby ammonia gas flows from the left through the tubing to gradually raise the pH of the supramolecular hydrogel D (the sample is wrapped in tin foil to minimise light exposure. (**b**) Representative photo demonstrating the change in turbidity within the hydrogel D, indicating the gradual disassembly of the supramolecular gel as ammonia diffuses through the sample to raise the pH (Red arrow shows a transparent area of solution and black arrow shows opaque gel). (**c**) CD spectra for gels at elevated pH, 1-1 Ag-GMP (red), A (green), and D (navy). (**d**) Rheology experiments demonstrating a gel retained after raising pH. Thin line plots indicate moduli prior to raising pH and the line and symbol plots post raising pH. For both, ▪ refers to G’ and • refers to G”, the storage modulus and viscous modulus, respectively.

Finally, the stimuli-responsive behaviour of the multicomponent hydrogels was demonstrated by selectively removing one of the gelator networks. The ability to selectively remove individual gelators is advantageous for modulating the mechanical properties of the gel and lowering the network density to affect the diffusion of cargo, for example, in therapeutic applications or stem cell differentiation in hydrogel-based tissue culture scaffold [20,21,77,78,79,80,81]. Given that the gelation of FY was strongly pH-responsive, we gradually increased the pH as an external stimulus to disintegrate the FY nanofilaments from hybrid hydrogel samples. To achieve this, ammonia gas was passively added to each of the gels individually by flowing the gas from an ammonia solution (ca. 18 M) into a vial containing the hydrogel (Figure 6a). This gradually increased the pH of the gel to a value of 8.5 over several hours, which was above the pKa of FY. As a result, deprotonation of the terminal carboxylic acid initiated the disassembly of the FY hydrogel filaments. Significantly, the guanine residues (pKa of 9.2) were not deprotonated at this pH because the most labile proton was already abstracted in the formation of the Ag-GMP dimer [24]. This was consistent with the control experiments with the GMP gels being intact after raising the pH to ca. 8.5. Thus, the multicomponent hydrogels prepared at an Ag:GMP molar ratio of 1:1 showed selective disassembly of the FY filaments at a pH of 8.5, leaving behind only the nucleotide-based hydrogel. As a result, the translucent multicomponent hydrogels (**A** and **B**) were transformed into transparent hydrogels similar to their parent Ag-GMP gel counterparts. The observations confirmed the orthogonal self-assembly of the two gelators into a network comprising two distinct types of supramolecular nanofilaments. This was analogous to interpenetrating polymer hydrogels, in which two networks co-exist [18,82,83].

Significantly, the CD spectroscopy (Figure 6c) of the ammonia-treated hybrid hydrogel samples **A** and **D** showed no signals related to the FY supramolecular structures and displayed peaks only characteristic of the nucleotide-based hydrogel, demonstrating that the self-assembled Ag-GMP nanofilaments remained intact. This was further supported by rheological studies (Figure 6d), which demonstrated that the hydrogel remaining after removal of the FY filaments exhibited comparable moduli to the single-component 1:1 Ag-GMP gel. Significantly, the yield stress increased for **A** (120%) and **B** (80%) after raising the pH compared to the original gels (ca. 55%). As a dense network structure was still observed by TEM after selective removal of the FY filaments (Figure S6), a dissolved FY gelator may have acted as a surfactant to improve the yield stress of the remaining Ag-GMP hydrogel. Conversely, the single-component Ag-GMP gel prepared at a 2:1 ratio did not disassemble at pH 8.5; however, the hybrid hydrogels (**E** and **F**) completely collapsed by increasing the pH. This indicated that at this ratio, separate filaments were not assembled, and therefore, it was not possible to selectively remove the FY nanofilaments without disrupting the Ag-GMP structures. This was further confirmed by CD spectroscopy (Figure 6c), which showed no peaks associated with the FY and Ag-GMP gelation after treatment at ca. pH 8.5. However, this does not mean that only co-assembled filaments are formed in these samples. Given the highly entangled network of supramolecular nanofilaments, it is also possible that the hybrid nanofilaments could lead to interpenetrated networks. For instance, tyrosine residues have previously been reported to intercalate within GMP stacks [46,47]. As a result, the disassembly of FY filaments could also influence the structural integrity of the Ag-GMP nanofilaments. The CD spectra for sample **D** showed peaks not associated with either FY or Ag-GMP gelation, confirming that both these filaments were disassembled. However, the formation of Ag(NH_3_)_2_^+^ complexes [84,85] in the system could also cause the disassembly of the Ag-GMP dimers. Thus, the peaks observed could instead be due to the formation of soluble G-quartets, which could then prevent re-assembly into a hydrogel [39,86]

## 3. Conclusions

In summary, we exploit a chemical signal generated during the silver ion-mediated dimerisation and gelation of guanosine molecules to trigger the assembly of an amino acid derivative. Interestingly, the Ag:GMP ratio in the hybrid hydrogels determines whether non-orthogonal assembly occurs. The results show that the Ag:GMP ratio has a significant impact on the supramolecular organisations such that a lower 1:1 ratio gives rise to more ordered assemblies in the presence of FY. Significantly, we show that the self-assembled nanofilaments of the amino acid derivative can be selectively disassembled by raising the pH, leaving a self-supporting supramolecular gel comprising Ag-GMP nanofilaments. Notably, the multicomponent hydrogels show improved mechanical properties (yield stress) compared to the single-component hydrogels. Overall, our strategy could be readily extended to design and construct novel hybrid supramolecular hydrogels comprising biologically relevant amino acids, peptide derivatives for biomedical applications, and soft-actuating materials.

## 4. Materials and Methods

Single-component hydrogels were prepared as follows. Silver nitrate (AgNO_3_) (Alfa Aesar, Heysham, UK) and guanosine 5′-monophosphate sodium salt (Na_2_GMP) (Sigma, St. Louis, MO, USA) were used as received, and all solutions were prepared using Milli-Q TM water. Ag-GMP hydrogels with AgNO_3_/GMP molar ratios of 1:1 and 2:1 were prepared at room temperature by adding an appropriate concentration of aqueous AgNO_3_ (25–100 mM) to an aqueous solution of Na_2_GMP (25–50 mM) whilst vortexing (1000 rpm) to aid dissolution of the silver salt for homogenous gelation, which occurred within a few hours.

N-Fluorenylmethyloxycarbonyl tyrosine (Fmoc tyrosine, FY) (Novabiochem, Merck, Darmstadt, Germany) was dissolved in 25 mM NaOH with the aid of sonication and vortexing. Aliquots of NaOH (1 M) were added to raise the pH of the solution to 9, which is above the pKa of the pendant carboxylic acid. The solutions were filtered through a 0.44 μm polyethersulfone syringe filter to remove any undissolved solid. Controlled acidification of the FY solutions was achieved by the addition of solid glucono-δ-lactone (GdL) (3 mg/mL), which slowly dissolved to give solutions with a final pH of ca. 4.5; as a consequence, single-component FY hydrogels were produced within a few hours.

To prepare multicomponent hydrogels, the stock FY solutions were used to dissolve desired amounts of GMP, which were followed by addition of an appropriate concentration of aqueous AgNO_3_. Typically, 25–100 mM AgNO_3_ was added to an aqueous solution containing a mixture of Na2GMP (25–50 mM) and FY (25–50 mM) whilst vortexing (1000 rpm) and left overnight to form hydrogels in the dark. The multicomponent hydrogels were prepared by maintaining the AgNO_3_:GMP molar ratios 1:1 and 2:1, whereas molar ratios of the GMP and FY were varied as shown below. The final concentration (mM) of each component is presented in brackets. AgNO_3_:GMP:FY molar ratios: (**A**) 1:1:1 (25 mM), (**B**) 1:1:0.5 (25, 25, 12.5 mM), (**C**) 0.5:0.5:1 (12.5, 12.5, 50 mM), (**D**) 2:1:1 (50, 12.5, 25 mM), (**E**) 2:1:0.5 (50, 25, 12.5 mM), and (**F**) 1:0.5:1 (25, 12.5, 25 mM).

## Figures and Tables

**Table 1:** A summary of compositions of gels prepared with their associated label and legend colour.

Samples	Ratios	Label	Type	Legend	pH	Result
FY			Solution	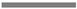	9.00	Solution
GMP			Solution	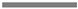	7.93	Solution
FY-GMP			Solution	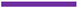	8.51	Solution
FY 25 mM GdL			Single component	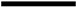	4.48	Gel
1:1 Ag:GMP			Single component	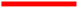	5.64	Viscous solution
2:1 Ag:GMP			Single component	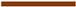	3.25	Gel
1:1 Ag:GMP	1:1:1 Ag:GMP:FY	A	Multicomponent	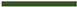	6.73	Gel
	1:1:0.5 Ag:GMP:FY	B	Multicomponent	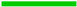	6.37	Gel
	0.5:0.5:1 Ag:GMP:FY	C	Multicomponent	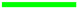	6.37	Gel
2:1 Ag:GMP	2:1:1 Ag:GMP:FY	D	Multicomponent	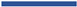	5.66	Gel
	2:1:0.5 Ag:GMP:FY	E	Multicomponent	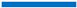	5.02	Gel
	1:0.5:1 Ag:GMP:FY	F	Multicomponent	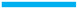	6.99	Gel

## Data Availability

The data generated or analysed during this study are available from the corresponding author on reasonable request.

## Supplementary Materials

The following are available online at https://www.mdpi.com/article/10.3390/gels7030146/s1. Figure S1: Photograph of single component hydrogels and control samples, Figure S2: TEM images of single component hydrogels, Figure S3: TEM images of multicomponent hydrogels, Figure S4: CD spectra of FY and GMP solutions and corresponding hydrogels, Figure S5: FTIR spectra of FY and GMP powders and corresponding lyophilised hydrogel powders, Figure S6: TEM images of multicomponent hydrogels, Table S1: Tgel-sol temperatures recorded for single and multicomponent hydrogels. Details of the characterisation methods and analysis of the FTIR data as well as further data are available in the Supplementary Materials section.
