# Supplementary material for: Stimuli-Responsive Nucleotide–Amino Acid Hybrid Supramolecular Hydrogels
Source: Gels. 2021 Sep 17;7(3):146. doi: 10.3390/gels7030146 (PMC8482081; doi:10.3390/gels7030146)
Supplement: Supplementary file 1 [file gels-07-00146-s001.zip › gels-1246838-supplementary.pdf]

Supplementary Materials

Stimuli-Responsive Nucleotide–Amino Acid Hybrid Supramolecular Hydrogels

Matthew Mulvee [1,2], Natasa Vasiljevic [2,3], Stephen Mann [1,2,*] and Avinash J Patil [1,*]

1 Centre for Organized Matter Chemistry, School of Chemistry, University of Bristol, Bristol BS8 1TS, UK; mattmulvee@gmail.com
2 Bristol Centre for Functional Nanomaterials, University of Bristol, Tyndall Avenue, Bristol BS8 1TL, UK; n.vasiljevic@bristol.ac.uk
3 School of Physics, University of Bristol, Bristol BS8 1TS, UK
* Correspondence: S.Mann@bristol.ac.uk (S.M.); Avinash.Patil@bristol.ac.uk (A.J.P.)

Characterization Methods

Differential scanning calorimetry (DSC) was carried out using a Mettler Toledo TGA/DSC1 Star System at a scan rate of 1 °C min^{-1} with a nitrogen flow of 25 mL min^{-1}. Rheometry was performed using a Malvern Kinexus fitted with a parallel plate geometry (gap width of 200 μm) at room temperature. Before rheology experiments, all hydrogel samples were aged for one day and were added to the rheometer using a spatula to minimise shear. The top plate is lowered, and the normal force is measured and allowed to reach equilibrium. Transmission electron microscopy (TEM) was performed in bright-field mode using a JEOL TEM 1400 electron microscope and JEOL TEM 2010 electron microscope operating at 120 keV. TEM samples were prepared by drop casting a dilute suspension of hydrogel (5 μL) onto carbon-coated copper TEM grids for three minutes and wicking excess fluid away using filter paper. All samples were left to dry overnight at room temperature. Cryo-TEM samples were imaged using an FEI Tecnai Twin Lens electron microscope fitted with an FEI Eagle 4 k × 4 k CCD camera and was operated at 200 keV. Attenuated total reflectance Fourier transform infrared spectroscopy (ATR-FTIR) was performed using a PerkinElmer Spectrum 100 FTIR spectrometer fitted with a universal attenuated total reflection accessory. The hydrogels were lyophilized by freezing in liquid nitrogen followed by freeze-drying for at least 24 h. CD spectra were recorded at room temperature using a JASCO J810 spectrometer through the two quartz plates. Samples were prepared by spreading hydrogels (ca. 25 μL) between two quartz plates to produce a homogeneous film and to reduce the scattering of light by the hydrogel samples. Experiments were conducted at a scan speed of 20 nm min^{-1} with ten accumulations.

Supplementary Figures

(a) **(i)** **(ii)** **(iii)** **(b)** **(i)** **(ii)**

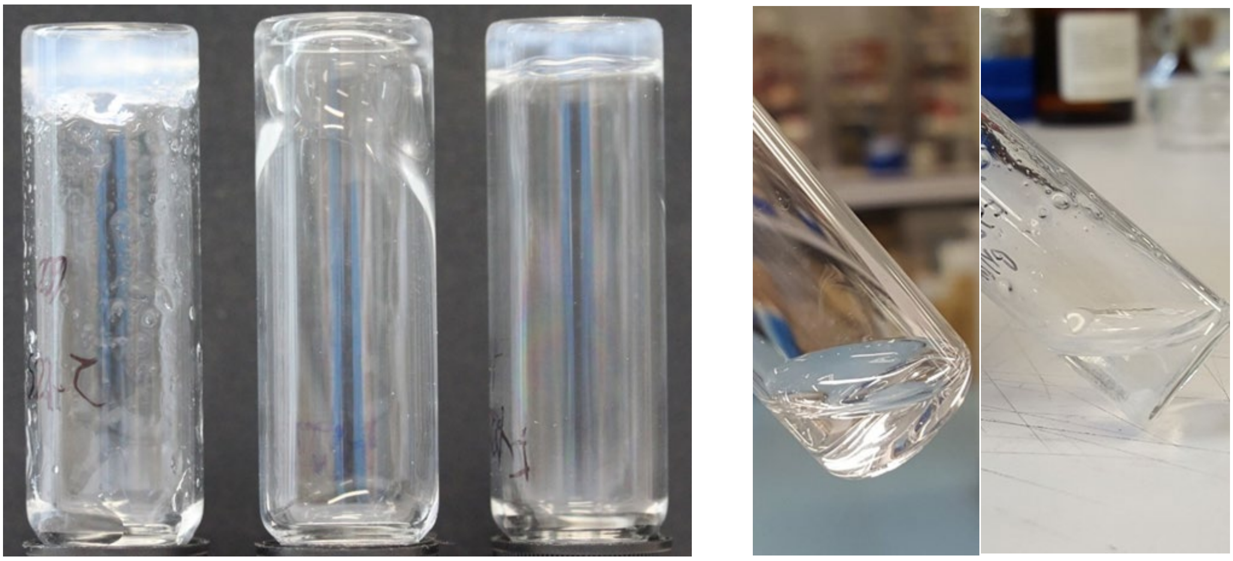

Figure S1. (**a**) Photographs of single component hydrogel samples; nucleotide hydrogel (i) 2:1 Ag:GMP, (ii) 1:1 Ag:GMP, and amino acid derivative FY gel (iii). (**b**) Photographs demonstrating that no gelation occurs unless all three components are present. (i) mixture of FY-GMP solution shows no gelation occurs without the addition of silver and (ii) no gelation upon the addition of silver to a FY solution. In this case, some precipitation was observed, possibly complexation between the silver salt and FY.

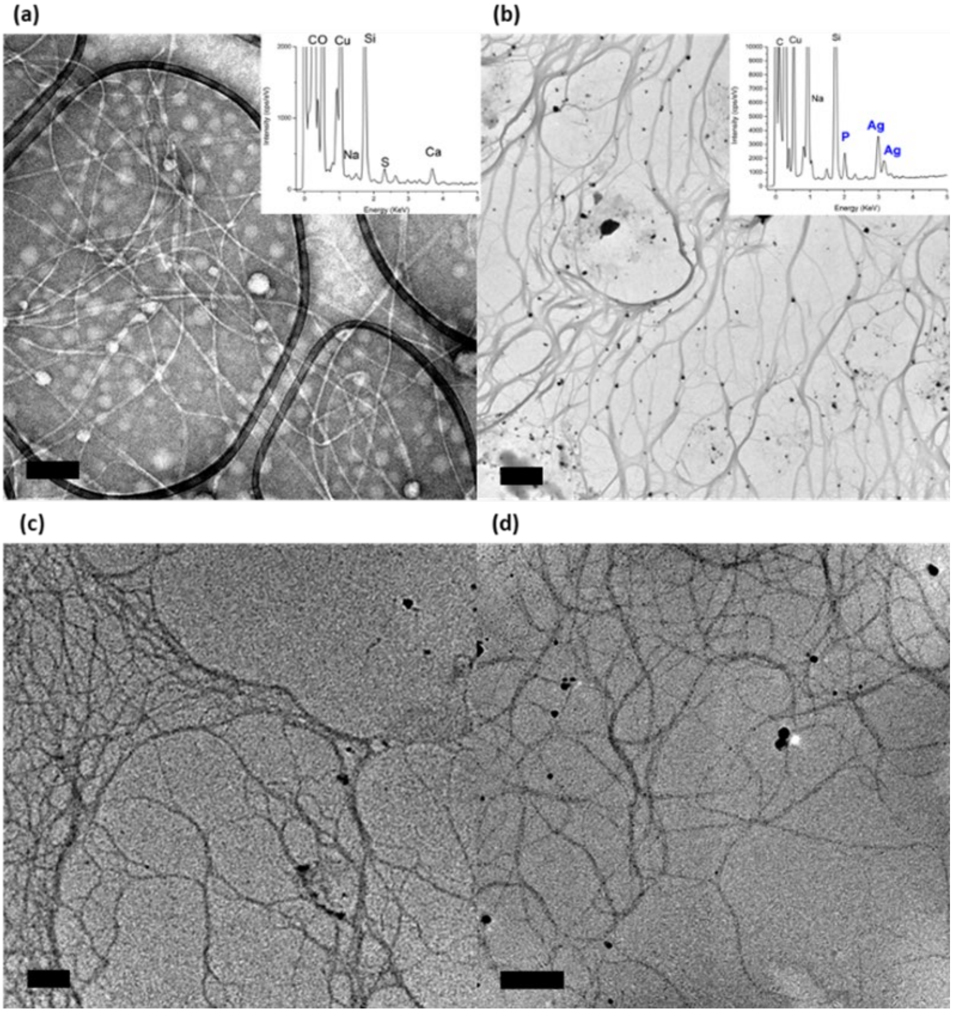

Figure S2. TEM images of single component hydrogels (**a**) FY nanofilaments, inset showing corresponding EDX, (**b**) negatively stained FY nanofilaments using uranyl acetate, (**c**) Ag-GMP gels 2:1 ratio, corresponding EDX (inset) showing presence of P and Ag (**d**) Ag-GMP gels prepared at 1:1 ratio. Scale bars a, c, and d = 100 nm, b = 1 μm.

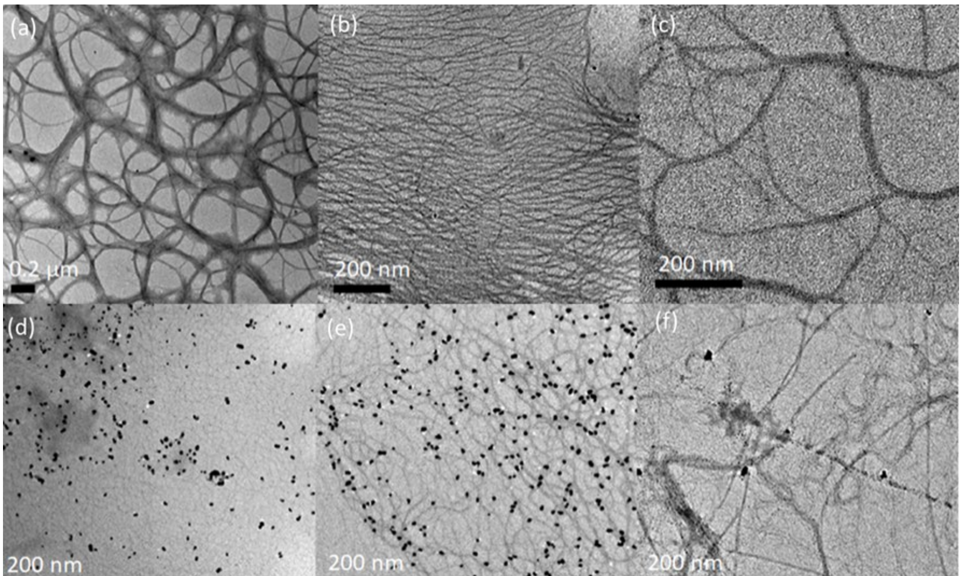

Figure S3. TEM images of multicomponent hydrogels samples prepared at 1:1 and 2:1 Ag-GMP stoichiometry and varying molar ratios of GMP and FY, (**a**) A, (**b**) B, (**c**) C, (**d**) D, (**e**) E and (**f**) F.

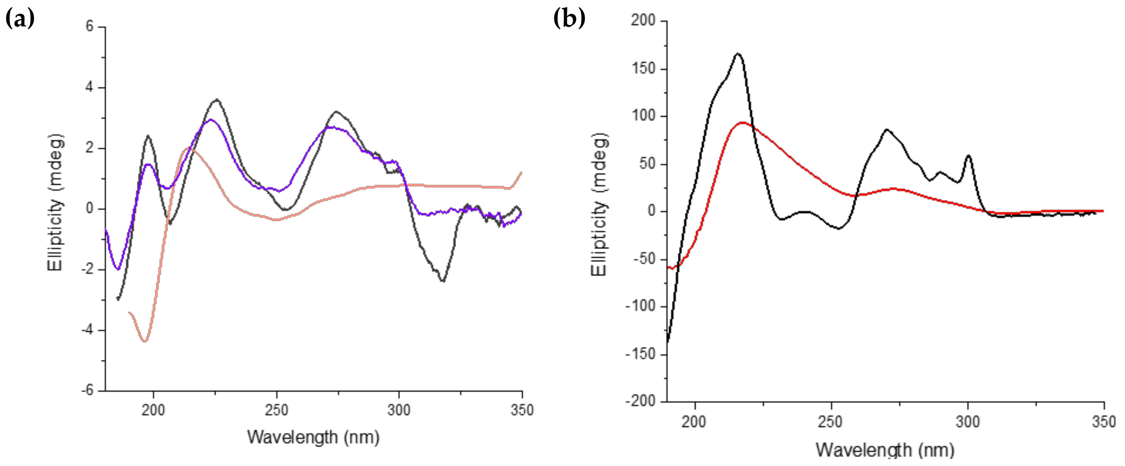

Figure S4. CD spectra of (**a**) **FY** and **GMP** gelator solutions and (**b**) single component gels. (**a**) FY (grey) and GMP (pink) and FY-GMP (purple). Single component hydrogels: (**b**) FY gel through hydrolysis of GdL (black) and Ag-GMP gel (red).

Further FT-IR analysis

The FT-IR spectra of the single-component samples were consistent with previous reports and a detailed analysis is useful to compare to the analysis of the hybrid gels summarised in the main text. Firstly, there are numerous peak shifts associated with the gelation of **GMP** compared to the **GMP** powder (Figure S4a,b). Most notably, the carbonyl vibration of the guanine residue shifts sustainably from its conjugated keto form (1669 cm^{-1}) [26,57,58], to a lower wavenumber in its enolate form (1606 cm^{-1} and 1605 cm^{-1}, for ratios 1:1 and 2:1, respectively), in line with previous reports [26,59]. This hypsochromic shift is indicative of a higher energy bond formation, relative to the conjugated keto carbonyl.

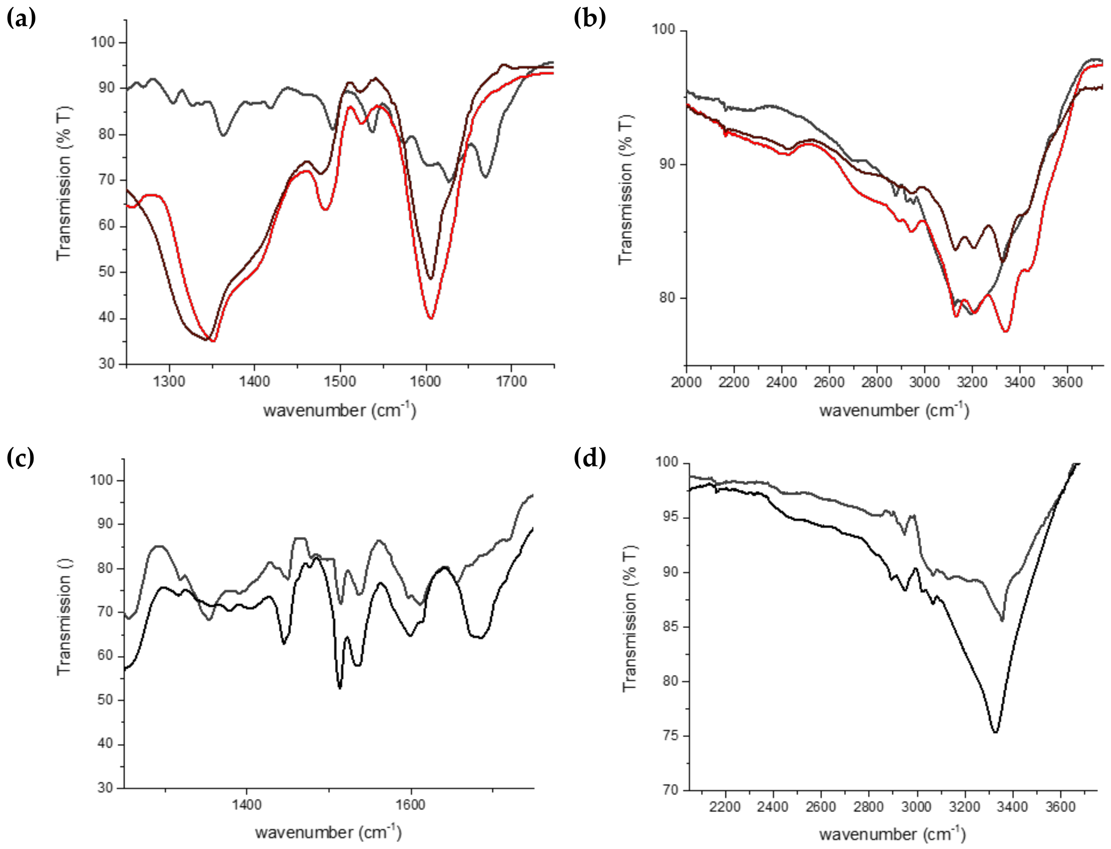

Figure S5. FT-IR spectra of (**a**) Fmoc-tyrosine powder (grey) (**b**) lyophilised powder of FY hydrogel prepared through hydrolysis of GdL (black), (**c**) GMP powder and lyophilised powders of Ag-GMP hydrogels; 1-1 (red) and, (**d**) 2–1 (maroon).

Many of the guanine ring stretches in this region observed in the powder are masked by this strong enol vibration. However, the C=C vibration (1537 cm^{-1}) is slightly shifted to a higher vibration (1524 cm^{-1}), possibly a consequence of disrupting the conjugated π-orbital system of the guanine ring [1]. Also, the NH_2 scissoring bend peak (1491 cm^{-1}) broadens and shifts to 1477 cm^{-1} upon gelation, indicative of becoming involved in hydrogen bonding [2,3]. In conjunction with this, at higher wavenumbers, are new peaks are observed at 3320 cm^{-1} and 3440 cm^{-1} characteristic of symmetric and asymmetric stretches, respectively, for hydrogen-bonded amine residues.[4] The broad peaks at 1321 and 1341 cm^{-1} for the 2:1 and 1:1 ratio, respectively, are associated with the full dissociated NO_3^- stretch, with the shoulder peak due to the vibrational mode for the ionic pair separated by one water molecule [5].

FTIR of **FY** (Figure S4c,d) shows several key differences between the gelled sample and the **FY** powder. These mainly originate from the Amide I regions, associated with carbonyl stretches, Amide II related to N-H bends and Amide A associated with hydrogen-bonded amine and hydroxyl groups. Gelation results in the disappearance of the 1656 cm^{-1}, associated with disordered or unstacked carbonyl groups [2,6]. This indicates more ordered assembly, which would be expected in the nanofilament formation. Gelation of **FY** is also marked by a slight bathochromic shift in the amide I region for the pendant carbonyl (1611 cm^{-1}) to 1614 cm^{-1} indicating more extended hydrogen bonding, as would be expected as electrostatic repulsion is lowered through protonation [2,7,8]. A broad non-Gaussian peak also emerges with two minima at 1675 and 1685 cm^{-1} characteristic of the carbamate carbonyl group, indicating that this group becomes involved in the hydrogen-bonding network as a result of gelation [2,6,9]. The former peak is associated with disordered carbamate hydrogen bonding and some disorder is to be expected for such small gelators [2,10,11]. The peak at 1599 cm^{-1} is the asymmetric stretching of the carboxylate anion indicating a proportion of the terminal carboxylic acid groups remain deprotonated [2,6,12]. Also, associated with gelation is a broad peak at 3321 cm^{-1} characteristic of hydrogen-bonded hydroxyl groups [13,14].

Table S1. Tgel-sol temperatures recorded from DSC thermograms of the single and multicomponent gels.

**Sample**	**$T_{gel-sol}$ (°C)**
FY	40.1
Ag:GMP 1:1	46.8
Ag:GMP 2:1	39.7
**A**	44.8
**B**	41.2
**C**	36.2 and 40.4
**D**	35.5 and 40.2
**E**	42.3 and 44.7
**F**	46.1

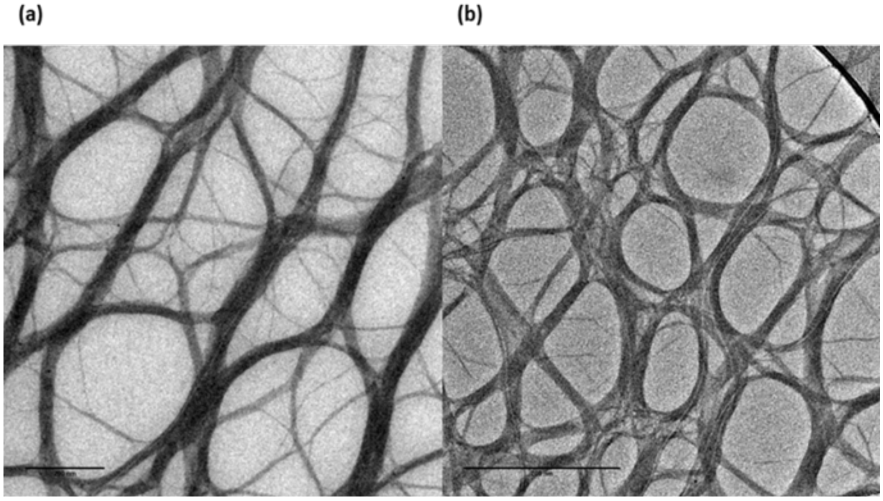

Figure S6. Representative TEM images of multicomponent hydrogel (Sample A, composition 1:1 Ag-GMP (25 mM) FY (12.5 mM) after the pH was raised to 8.7, clearly demonstrating that filaments are intact. Scale bars = 200 nm.

References

1. Tu, T.; Reinosa, J.A. The interaction of silver ion with guanosine, guanosine monophosphate, and related compounds. Determination of possible sites of complexing. *Biochemistry* **1966**, *5*, 3375–3383.
2. Fleming, S.; Frederix, P.W.J.M.; Ramos Sasselli, I.; Hunt, N.T.; Ulijn, R.V.; Tuttle, T. Assessing the utility of infrared spectroscopy as structural diagnostic tool for β sheets in self-assembling aromatic peptide amphiphiles. *Langmuir* **2013**, *29*, 9510–9515.
3. Hashemnejad, S.M.; Kundu, S. Probing gelation and rheological behavior of a self-assembled molecular gel. *Langmuir* **2017**, *33*, 7769–7779.
4. Zhizhina, G.P.; Oleinik, E.F. Infrared spectroscopy of nucleic acids. *Russ. Chem. Rev.*, **1972**, *41*, 258–280.
5. De La Fuente, M.; Hernanz, A.; Navarro, R. IR and Raman studies on the interactions of 5′-GMP and 5′-CMP phosphate groups with Mg (II), Ca (II), Sr (II), Ba (II), Cr (II), Co (II), Cu (II), Zn (II), Cd (II), Al (III) and Ga (III). *J. Biol. Inorg. Chem.* **2004**, *9*, 973-986. DOI:10.1007/s00775-004-0593-5.
6. Fleming, S.; Debnath, S.; Frederix, P.W.J.M.; Hunt, N.T.; Ulijn, R.V. Insights into the coassembly of hydrogelators and surfactants based on aromatic peptide amphiphiles. *Biomacromolecules* **2014**, *15*, 1171–1184.
7. Sukul, P.K.; Malik, S. Removal of toxic dyes from aqueous medium using adenine based bicomponent hydrogel. *RSC Adv.* **2013**, *3*, 1902–1915.
8. Baral, A.; Basak, S.; Basu, K. ; Dehsorkhi, K.; Hamley, I.W.; Banerjee, A. Time dependent gel to gel transformation of a peptide based supramolecular gelator. *Soft Matter* **2015**, *11*, 4944–4951.
9. Singh, V.; Snigdha,K.; Singh, C.; Sinha, N.; Thakur, A.K. Understanding the self-aseembly of FMOC-phenylalanine to hydrogel. *Soft Matter* **2015**, *11*, 5353–5364.
10. Fleming, S.; Debnath, S.; Frederix, P.W.J.M.; Tuttle, T.; Ulijn, R.V. Aromatic peptide amphiphiles:significance of the Fmoc moiety. *Chem. Commun. (Camb).* **2013**, *49*, 10587–10589.
11. Wang, J.; Liu, K.; Xing, R.; Yan, X. Peptide assembly:thermodynamics and kinetics. *Chem. Soc. Rev.*, 2016, **45**, 5589–5604.
12. Abul-Haija, Y.M.; Roy, S.; Frederix, P.W.J.M.; Javid, N.; Jayawarna, V.; Ulijn, R.V. Biocatalytically triggered co-assembly of two component core-shell nanofibers. *Small* **2014**, *10*, 973–979.
13. Metreveli, N.O.; Jariashvili, K.K.; Namicheishvili, L.O.; Svintradze, D.V; Chikvaidze, E.N.; Sionkowska, A.; Skopinska, J. Uv-vis and FTIR spectra of ultraviolet irradiated collagen in the presence of antioxidant ascorbic acid. *Ecotoxicol. Environ. Saf.* **2009**, *73*, 448–455.
14. Gerothanassis, P.; Birlirakis, N.; Sakarellos, C.; Marraud, M. Solvation state of the Tyr side chain in peptides. An FTIR and ^{17}O NMR approach. *J. Am. Chem. Soc.* **1992**, *114*, 9043–9047.
